# The prognostic impact of decreased pretreatment haemoglobin level on the survival of patients with lung cancer: a systematic review and meta-analysis

**DOI:** 10.1186/s12885-018-5136-5

**Published:** 2018-12-10

**Authors:** Yaqi Huang, Siqi Wei, Nan Jiang, Lijuan Zhang, Siyuan Wang, Xiaona Cao, Yue Zhao, Peiguo Wang

**Affiliations:** 1 School of Nursing, Tianjin Medical University, Tianjin, 300070 China; 2 School of Nursing, Liaoning University of Traditional Chinese Medicine, Liaoning, China; 3 Department of Radiotherapy, Tianjin Medical University Cancer Institute and Hospital, National Clinical Research Center for Cancer, Tianjin, 300060 China

**Keywords:** Lung cancer, Haemoglobin, Prognosis, Meta-analysis

## Abstract

**Background:**

Many studies have reported the prognostic value of haemoglobin level for cancers. Whereas the prognostic impact of decreased pretreatment haemoglobin level on the survival of patients with lung cancer remains controversial, herein, a systematic review and meta-analysis were conducted to investigate whether a decreased haemoglobin level before treatment is a significant predictor of survival in patients with lung cancer.

**Methods:**

We performed a systematic review and meta-analysis of observational studies to evaluate the prognostic impact of a decreased haemoglobin level on the survival of patients with lung cancer. Relevant studies were retrieved from databases including PubMed, Embase, Web of Science and the Cochrane Library. Reference lists were hand-searched for potentially eligible studies. The Newcastle-Ottawa scale was used to assess the quality of included studies. Observational studies were included if they provided sufficient information for the extraction of the pooled hazard ratios (HR) and 95% confidence intervals (95% CI) for overall survival, disease-free survival, relapse-free survival, progression-free survival, event-free survival and time to progression. Subgroup analysis, meta-regression and sensitivity analyses were applied to explain the heterogeneity.

**Results:**

Fifty-five articles involving a total of 22,719 patients were obtained to evaluate the correlation between haemoglobin level and survival. The results indicated that decreased haemoglobin level was significantly associated with poor overall survival of patients with lung cancer (HR 1.51, 95% CI 1.42–1.61), both in non-small cell lung cancer (HR 1.57, 95% CI 1.44–1.72) and in small cell lung cancer (HR 1.56, 95% CI 1.21–2.02). We also found that the lower the haemoglobin level, the shorter was the overall survival of patients with lung cancer (HR 1.11, 95% CI 1.06–1.16). However, the relationship between decreased haemoglobin and relapse-free survival was not significant (HR 1.37, 95% CI 0.91–2.05).

**Conclusion:**

A decreased pretreatment haemoglobin level among patients with lung cancer is a prognostic factor of poor survival that can serve as an important indicator in survival prediction, risk stratification and treatment selection. In clinical practice, more attention should be paid to monitoring pretreatment haemoglobin levels among patients with lung cancer.

**Electronic supplementary material:**

The online version of this article (10.1186/s12885-018-5136-5) contains supplementary material, which is available to authorized users.

## Background

Lung cancer is the most prevalent cancer and the leading cause of cancer-related death in both men and women [1, 2]. Although integrated treatment strategies and multidisciplinary nursing interventions based on surgery, radiotherapy and chemotherapy have provided improvements in the survival of patients with lung cancer, more effective prognostic factors should be identified to guide therapy and assess disease progression in individuals. In previous studies, the tumour-node-metastasis (TNM) staging system and tumour markers have made great contributions to the prediction of clinical outcomes, though most of these markers are clinicopathological parameters determined after surgery and are associated with high costs. Thus, it is important to detect new predictors to satisfy clinical requirements [3, 4].

Decreased haemoglobin (Hb) is the most commonly observed haematological abnormality in patients with cancers; it is induced by the direct or the indirect effects of malignancy or its treatment [5]. The National Comprehensive Cancer Network considered that Hb levels less than 11 g/dl can be diagnostic of cancer-related decreased Hb [6]. The mechanism of Hb degradation in lung cancer is complex. Blood loss, haemolysis, marrow infiltration and nutritional deficiencies may all be responsible for the development of Hb decline. Cancer-stimulated production of inflammatory cytokines (e.g. TNF-α, IL-1, IL-6, INF-γ) can inhibit erythropoiesis resulting in Hb reduction [7, 8]. The Hb level is a convenient and well-known parameter in clinical practice. An increasing body of evidence indicates that decreased Hb is related to poor prognosis in cancers [4, 9, 10]. However, the prognostic value of Hb level in patients with lung cancer has not been well confirmed. Numerous previous studies that have examined this relationship provide conflicting results [11–14]. Some studies showed that overall survival (OS) was significantly shorter in lung cancer patients with a low Hb level before treatment [11, 12], while some showed that the correlation between low Hb level and shorter OS was not significant [13, 14]. Therefore, in this study, a meta-analysis was conducted to determine the prognostic value of decreased Hb level in patients with lung cancer.

## Method

### Search strategy

Relevant studies that referred to the prognostic value of the Hb level in patients with lung cancer were identified by searching several databases up to November 2017, including PubMed, Embase, Web of Science and Cochrane Library. We used the following terms as MeSH terms and free-text terms (‘lung neoplasm’, ‘lung cancer’, ‘lung carcinoma’, ‘lung tumor’), (‘hemoglobin’, ‘Hb’ ‘hemoglobinometry’, ‘anemia’) and (‘mortality’, ‘prognosis’, ‘prognostic’, ‘predict’, ‘predictive’, ‘predictor’, ‘survival’, ‘outcome’); only studies published in English were retrieved. The references of candidate studies were also reviewed.

### Inclusion and exclusion criteria

The identified studies were independently selected by two reviewers following the inclusion and exclusion criteria below. Disagreements were discussed in a group to reach consensus. Studies were included if they met the following criteria: (1) The study population was patients who were diagnosed with lung cancer; (2) The serum Hb level was measured before treatment; (3) The relationship between the Hb level and survival was provided; and (4) A univariate Log-rank test or multivariate Cox proportional hazards model was used for statistical analysis; only observational studies were selected. Studies were excluded if they met one of the following criteria: (1) Studies were not published in English; (2) The full-text could not be obtained; (3) Data were not sufficient to extract the hazard ratio (HR) and 95% confidence interval (CI); and (4) Survival data were only provided as Kaplan-Meier curves; repeated studies or duplicate data were excluded. If one author reported the same population in different articles, only the most detailed one was included.

### Quality assessment

Two reviewers evaluated the quality of each study independently. The Newcastle-Ottawa scale (NOS) was used to assess the quality of included studies. The scale contains 8 items in 3 dimensions (selection, comparability and outcome) [15]. The assessment was carried out by awarding stars for high-quality studies, ranging from zero up to nine stars. A score of more than 6 stars indicates a high quality [16].

### Data extraction

Two reviewers extracted data from the eligible studies independently. Any discrepancy in data extraction was resolved through a cross-check and discussion. The primary data extracted were HR for poor prognosis with 95% CI, or the data necessary to calculate the HR and 95% CI. HRs from multivariate analyses were extracted if both univariate and multivariate analyses were provided. The characteristics of the studies and patients were collected, including the first author, year published, country, number of patients, gender, mean or median age of patients, duration of follow-up, subtype of lung cancer, stage of the tumour, treatment modalities, Hb cut-off value, indicator of survival analysis, and statistical methods.

### Statistical analysis

All statistical analyses were performed with Stata statistical software, version 15.0 (Stata Corp LLC, College Station, TX, USA). The association between Hb level and prognosis of patients with lung cancer was estimated by calculating the pooled HR and 95% CI. We used the random-effect model to combine the effective value based on heterogeneity [17]. A *p* value < 0.05 was considered to be significant in all statistical tests. HR > 1 indicated a negative prognosis in patients with a low Hb level. The heterogeneity of the pooled HRs was assessed using the Cochran’s *Q* test and *I*^*2*^ test, and a *p* value less than 0.05 or an *I*^*2*^ more than 50% was considered to be statistically significant [18]. To explain heterogeneity, subgroup analyses were performed by stratifying the included studies by lung cancer subtype and statistical method. To further explore the sources of heterogeneity, meta-regression analyses were conducted. We also performed sensitivity analyses by deleting one study at a time to estimate the contribution of included studies to heterogeneity. Egger’s indicator test and Begg’s funnel plot were applied to scrutinize publication bias [19, 20].

## Result

### Study retrieval

**Fig. 1:**
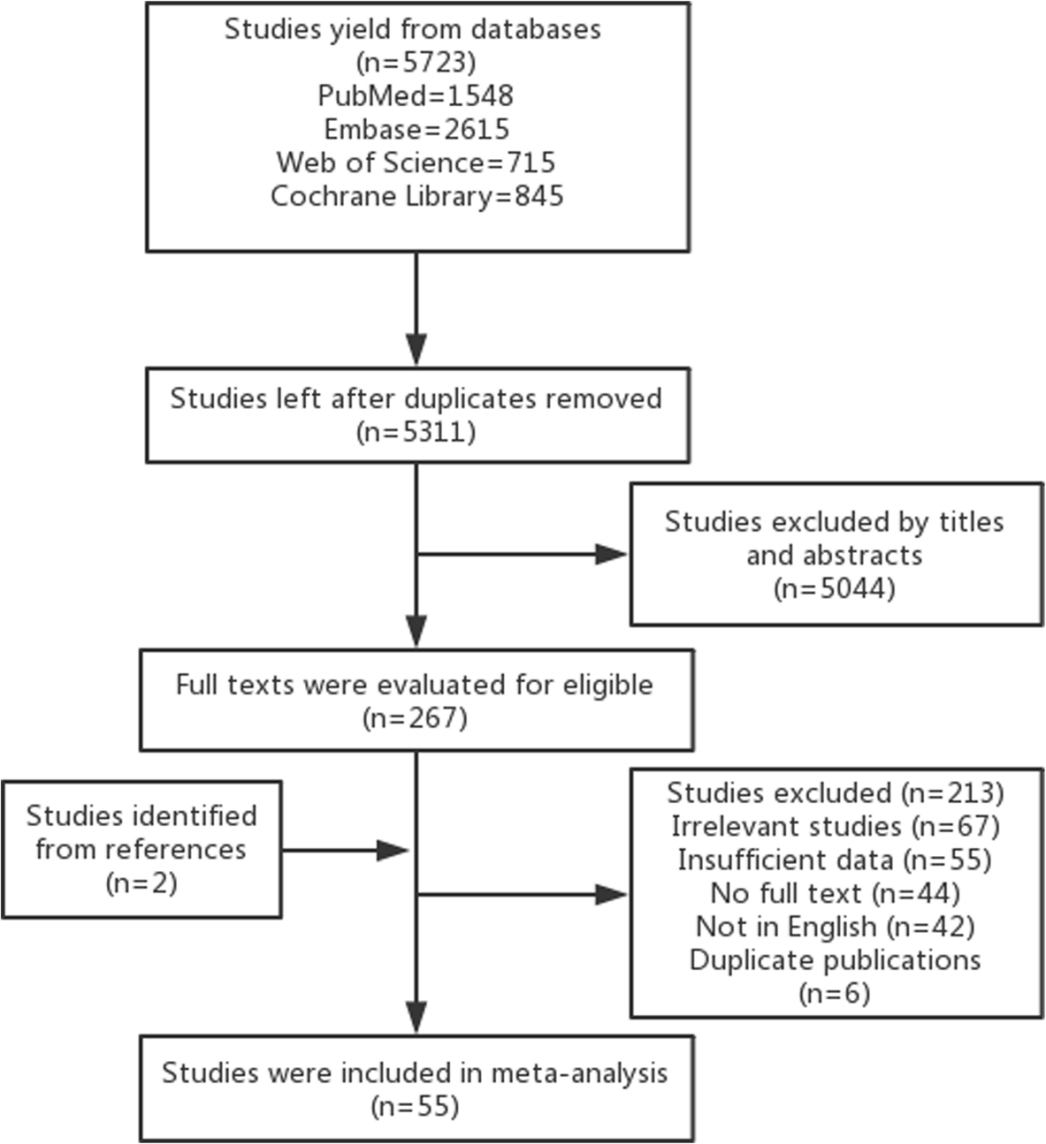
Flow diagram following the searching strategy for studies included in this meta-analysis

A total of 5723 citations were retrieved using the search strategy described above. Four hundred twelve duplicate records were removed. After screening and scanning the titles and abstracts of the publications, 5044 studies were excluded for being reviews, animal experiments, case reports, letters, comments, drug clinical trials, or otherwise irrelevant to our studies. After reviewing the full texts of 267 candidate studies, 213 articles were excluded according to the criteria above. Of these, 67 articles were excluded for being irrelevant to our study. For example, one study investigated the effect of abnormal Hb level (< 12 g/l or > 18 g/l) on the prognosis of lung cancer instead of investigating decreased Hb specifically, and some studies focused on the relationship between outcomes and decreased Hb during therapy rather than pretreatment levels. Fifty-five articles were excluded for reporting insufficient data to calculate HR, 44 articles for not having full text available, 42 for being published in other languages, and 5 for being duplicate publications. Two additional non-duplicate studies were identified from study reference lists. Finally, a total of 56 studies including 22,719 patients were included in this meta-analysis. The detailed search process is shown in Fig. 1.

### Study characteristics

The main characteristics of all eligible studies are shown in Table 1**.** Forty-eight studies were analysed with decreased Hb level as the categorical variable, 38 of which provided data on the relationship between OS and Hb in patients with non-small cell lung cancer (NSCLC); 6 studies were conducted in patients with small cell lung cancer (SCLC); and 4 studies included both patients with NSCLC and SCLC. Eight of the 56 included studies were analysed with pretreatment Hb as a continuous variable. Moreover, 3 studies were also available for disease-free survival (DFS), relapse-free survival (RFS) and progression-free survival (PFS) extraction, respectively. Only one study reported the relationship between the Hb level, event-free survival (EFS) and time to progression (TTP).

**Table 1:** Characteristics of studies included for meta-analysis

Author	Year	Country	Subtype	Tumor stage	Sample size	Median Age(years)	Gender (M/F)	Treatment modality	Follow up (months)	Survival analysis	Cut-off value (g/dl)	Analysis	Quality^a^
Osterlind, K [21]	1986	Denmark	SCLC	NR	778	NR	NR	Chemoradiotherapy	NR	OS	12	MV	6
Albain, K S [22]	1991	USA	NSCLC	NR	1925	NR	77%/23%	Chemotherapy	NR	OS	11	MV	5
Takigawa, N [23]	1996	Japan	NSCLC	Advanced	186	68	134/51	Chemoradiotherapy	NR	OS	11	MV	7
Wigren, T [24]	1997	Finland	NSCLC	Mix	502	65	459/43	Radiotherapy	48	OS	12.5	MV	6
Ohlhauser, C [25]	1997	Germany	NSCLC	Mix	456	65.5	391/65	Radiotherapy	NR	OS	12.7	MV	6
Jazieh, A R [26]	2000	USA	NSCLC	Early	454	67	410/44	Surgery	28	OS,EFS	10	MV	5
Rzyman, W [27]	2003	Poland	NSCLC	Mix	493	59.7	493/100	Surgery	NR	OS	12	MV	5
Bremnes, R M [28]	2003	Norway	SCLC	Limited: 214 Extensive: 222	436	64	280/156	Chemoradiotherapy	>60	OS	Male: 13 Female: 11.5	MV	5
Langendijk, H [29]	2003	Netherland	NSCLC	Mix	529	68	87%/13%	Radiotherapy	>24	OS	Continuous	MV	6
Tammemagi, C M [30]	2003	USA	LC	NR	NR	NR	NR	Mix	29.7	OS	NR	MV	5
Yovino, S [31]	2005	USA	NSCLC	Early	82	68	48/34	Surgery	20.8	OS,RFS	12	MV	7
Berardi, R [32]	2005	Italy	NSCLC	Mix	439	68	374/65	Surgery	27	OS	10	MV	7
Pradier, O [33]	2005	Germany	NSCLC	Advanced	56	NR	44/12	Radiotherapy	NR	OS	11.6	UV	7
Aoe, K [34]	2005	Japan	LC	Mix	611	64	482/129	NR	NR	OS	Male: 13 Female: 12	MV	7
Mohan, A [35]	2006	India	SCLC	Limited: 27.6% Extensive: 72.4%	76	54.9	84.2%/5.8%	Chemoradiotherapy	NR	OS	12.8	MV	5
Mandrekar, S J [36]	2006	USA	NSCLC	Advanced	1053	63.3	NR	NR	NR	OS, TTP	Male: 13.2 Female: 11.5	MV	5
Laurie, SA [37]	2006	Canada	SCLC	Limited	130	62	63/67	Chemoradiotherapy	NR	OS, PFS	10	MV	6
Paul, I [38]	2006	UK	NSCLC	Mix	42	68.1	35/7	Surgery	55.2	OS	Continuous	MV	6
Gauthier, I [39]	2007	Canada	NSCLC	Early	476	61.3	311/165	Chemotherapy Surgery	NR	OS	12	MV	5
Ademuyiwa, F O [40]	2007	India & USA	NSCLC	Advanced	2013	NR	134/69	Chemoradiotherapy	25.6	OS	Continuous	MV	6
Panagopoulos, ND [41]	2008	Greece	NSCLC	Mix	331	64	295/36	Surgery	27.2	OS	12	MV	7
Park, M J [42]	2008	Korean	NSCLC	NR	358	NR	NR	Chemotherapy	NR	OS	10	MV	5
Jacot, W [43]	2008	France	NSCLC	Mix	301	63	242/59	Mix	20.8	OS	11	MV	6
Florescu, M [44]	2008	Canada	NSCLC	Advanced	485	NR	313/72	Chemotherapy	NR	OS	Male: 13.6 Female: 12	MV	5
Stinchcombe, T E [45]	2009	USA	NSCLC	Advanced	331	NR	218/113	Chemoradiotherapy	88	OS	13	MV	6
Garrido, P [13]	2009	Spain	NSCLC	Advanced	139	NR	127/12	Chemoradiotherapy	23	OS	12	MV	6
Belbaraka, R [46]	2010	France	NSCLC	Advanced	45	58.5	30/15	Chemotherapy	NR	OS	Male: 11.5 Female: 13	MV	7
Qiu MZ [47]	2010	China	NSCLC	Mix	430	59	310/120	Mix	31	OS	11	UV	6
Ovcaricek, T [48]	2010	Slovenia	NSCLC	Mix	53	65	40/13	Chemotherapy	NR	PFS	Continuous	MV	6
Yi, S Y [49]	2011	Korea	NSCLC	Advanced	191	72	NR	Chemotherapy	NR	OS	12	MV	6
Castro, J G [50]	2011	Brazil	NSCLC	Advanced	142	63	95/47	Chemotherapy	NR	OS	12	UV	5
Kishida, Y [14]	2011	Japan	NSCLC	Advanced	86	65	72/14	Chemoradiotherapy	20	OS	12	UV	7
Janku, F [51]	2011	USA	NSCLC	Mix	85	62	51/34	Chemotherapy+ targeted	NR	OS	12	UV	5
Gioulbasanis, I [52]	2011	Greece	LC	NR	115	66	101/14	Chemotherapy	38.2	OS	Continuous	UV	7
Hsu C L [53]	2012	China	NSCLC	Advanced	144	39.1	70/74	Chemoradiotherapy	NR	OS	11	MV	6
Holgersson G [54]	2012	Sweden	NSCLC	Mix	833	NR	NR	Mix	NR	OS	11	MV	5
Ng T [55]	2012	USA	NSCLC	Early	361	NR	161/200	Surgery	48	OS, DFS	Male: 13 Female: 12	MV	7
Wu, C [56]	2012	China	SCLC	Extensive	200	NR	174/26	Chemoradiotherapy	NR	OS	NR	MV	6
Kiely, B E [57]	2013	Australia	NSCLC	Advanced	244	64	146/98	Chemotherapy	21	OS	12	UV	5
Tas, F [58]	2013	Turkey	LC	Mix	100	59	91/9	Chemotherapy	5	OS	12	UV	5
Qu, X [59]	2014	China	NSCLC	Mix	649	58.9	456/193	Surgery	43	OS, RFS	14.6	MV	6
Smith, M O [60]	2014	UK	NSCLC	Mix	563	68.5	305/258	Surgery	NR	OS	13.1	MV	5
Kacan, T [61]	2014	Turkey	NSCLC	Mix	299	61	270/29	Mix	NR	OS	12	MV	5
Strouse, C S [12]	2014	USA	NSCLC	Advanced	2845	NR	NR	Chemotherapy	NR	OS	NR	MV	5
Oguz, A [62]	2014	Turkey	NSCLC	Advanced	186	63	161/25	NR	NR	OS	Continuous	MV	5
Crvenkova, S [63]	2015	Republic of Macedonia	NSCLC	Advanced	85	58.2	75/10	Chemoradiotherapy	36	OS	12	UV	6
Wu, X Y [64]	2015	China	NSCLC	Advanced	186	NR	NR	Chemoradiotherapy	>36	OS	12	UV	6
Imai, H [65]	2015	Japan	NSCLC	Advanced	159	64	126/33	Radiotherapy	NR	OS	Continuous	MV	5
Xie, D [66]	2015	China	SCLC	Limited:555 Extensive: 383	938	68	500/438	Mix	10.8	OS	12	MV	6
Abazari M [67]	2015	Iran	LC	Mix	355	63.5	256/99	Mix	NR	OS	14	UV	5
Cata, J P [68]	2016	USA	NSCLC	Early	861	65.29	394/467	Surgery	108.28	OS, RFS	Male: 13 Female: 12	MV	6
Shaverdian, N [69]	2016	USA	NSCLC	Early	110	76	NR	radiotherapy	28.9	OS,DFS	12	MV	5
Lin, Y [11]	2016	China	NSCLC	Mix	69	56	54/15	Mix	NR	OS,DFS	Male: 12 Female: 11	MV	6
Park S [70]	2016	Korea	NSCLC	Mix	630	64	236/394	Chemotherapy	NR	OS, PFS	Male: 13 Female: 12	UV	5
Shaverdian, N [69]	2016	USA	NSCLC	Early	147	NR	NR	Radiotherapy	28.9	OS, DFS	Continuous	MV	6
Minami, S [71]	2016	Japan	NSCLC	Advanced	103	69.5	85/18	Chemotherapy	NR	OS	Continuous	MV	6
Lee S [72]	2017	Korea	NSCLC	Advanced	135	NR	78/57	Korean medicine	NR	OS	Male: 13 Female: 12	UV	5

Abbreviations: *NSCLC* non-small cell lung cancer, *SCLC* small cell lung cancer, *LC* lung cancer, *M/F* male/female, *NR* not reported, *OS* overall survival, *DFS* disease-free survival, *RFS* relapse-free survival, *PFS* progression-free survival, *EFS* event-free survival, *TTP* time to progression, *MV* multivariate, *UV* univariate

^a^The quality of studies was assessed by Newcastle-Ottawa scale

### OS and decreased Hb

**Fig. 2:**
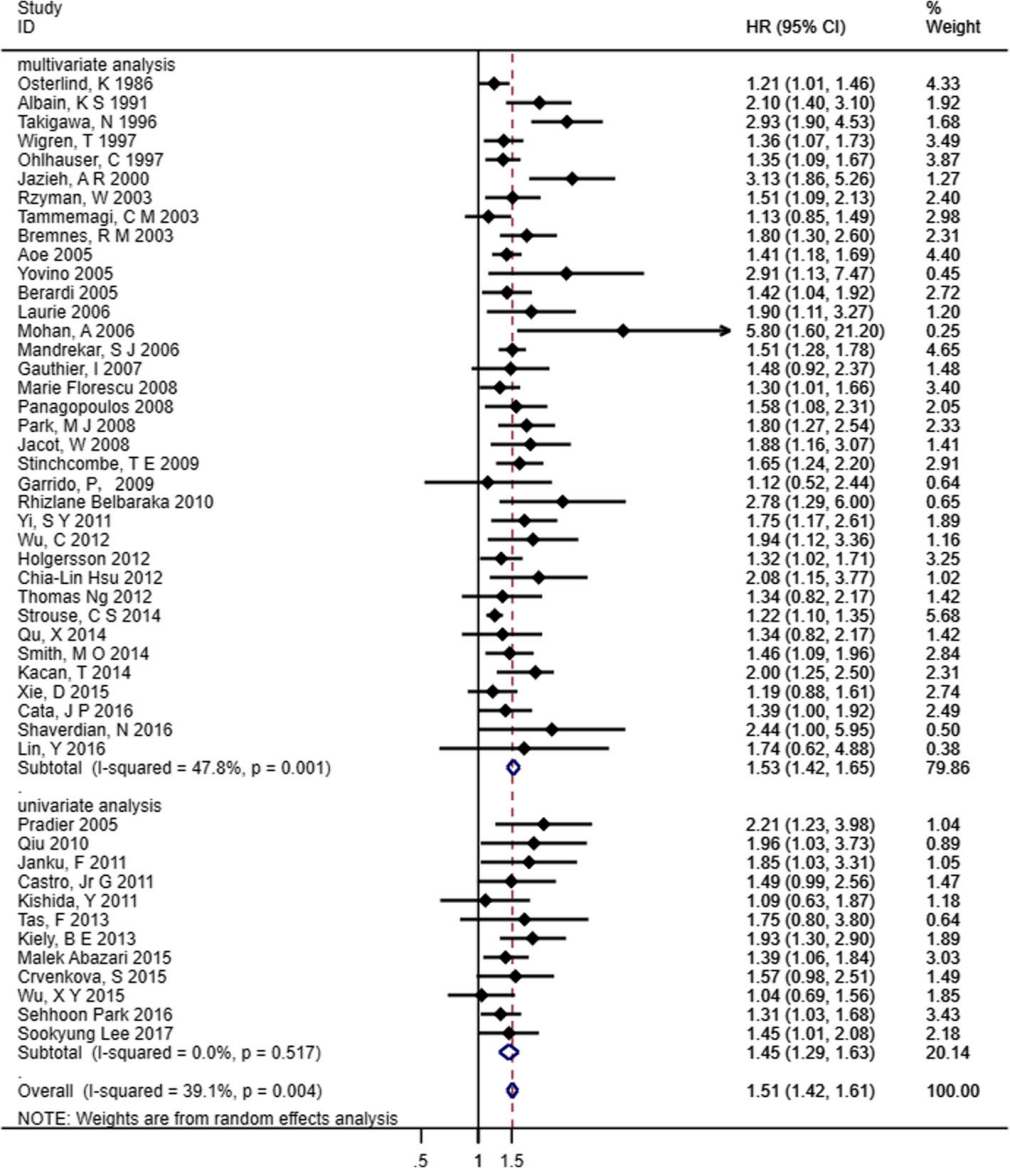
Forest plot and pooled HR and 95% CI for OS in patients with lung cancer: pretreatment decreased Hb vs. normal Hb. The pooled HR for OS showed that the patients with pretreatment decreased Hb level possessed a worse outcome in OS. *HR* hazard ratios, *OS* overall survival, *CI* confidence interval, *Hb* hemoglobin

Forty-eight articles with data on overall survival and decreased Hb (categorical variable: decreased Hb vs. normal Hb) were included in the pooled analysis. There was significant heterogeneity among these studies (*I*^*2*^ = 39.1%, *p* = 0.004), and thus, the random effect model was employed to calculate the pooled HR and its 95% CI. Lower Hb was significantly correlated with poor OS (HR 1.51, 95% CI 1.42–1.61). For further exploration, subgroup analyses were conducted. Forty-eight studies were re-classified by “analysis method”. In univariate analysis studies, there appeared to be no heterogeneity among HRs (*I*^2^ = 0.0%, *p* = 0.517), and we found that decreased Hb was a negative prognostic factor for OS (HR 1.45, 95% CI 1.29–1.63). Similarly, as shown in multivariate analyses, 36 studies also indicated that decreased pretreatment Hb predicted a significantly worse OS in patients with lung cancer (HR 1.53, 95% CI 1.42–1.65) (Fig. 2).

**Fig. 3:**
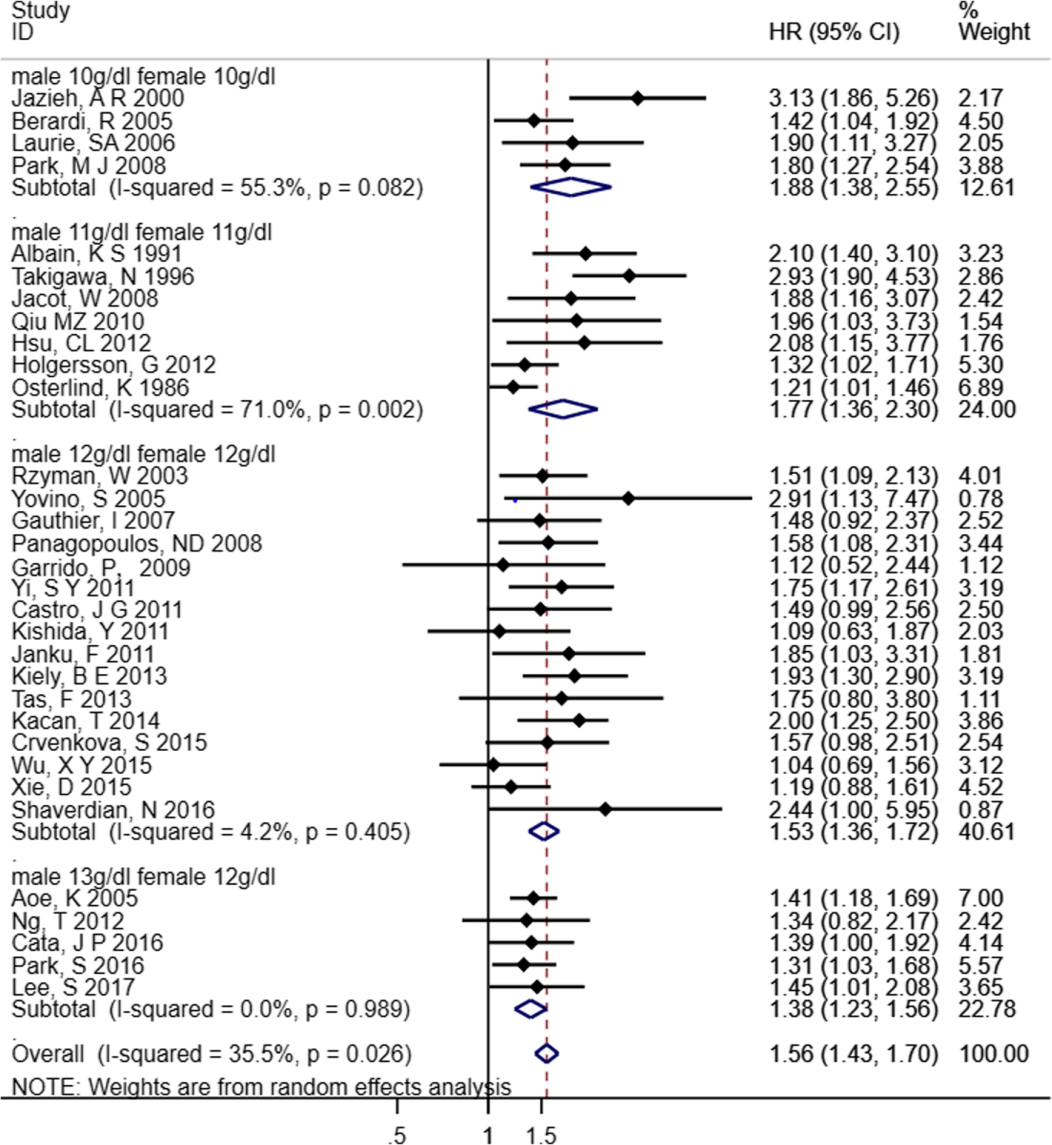
Forest plot and pooled HR and 95% CI for OS in patients with lung cancer: pretreatment decreased Hb vs. normal Hb with different Hb cut-off values. The pooled HR for OS showed the pretreatment decreased Hb was an independent prognostic factor of survival in patients with lung cancer. *HR* hazard ratios, *CI* confidence interval, *Hb* hemoglobin

Cut-off values of 10 g/dl, 11 g/dl, and 12 g/dl, along with gender-specific values of 13 g/dl (males) and 12 g/dl (females), were mostly used in the included studies. We divided these studies into 4 subgroups based the Hb cut-off values used: 10 g/dl, 11 g/dl, 12 g/dl and gender-specific (male 13 g/dl, female 12 g/dl). In total, the HRs of 32 studies were pooled in this meta-analysis. The results showed that decreased Hb before treatment was a significant predictor of OS in patients with lung cancer (HR 1.56, 95% CI 1.43–1.70). Although the heterogeneity was still significant in the 11 g/dl group (*I*^*2*^ = 71%, *p* = 0.002), there was no significant heterogeneity overall or in the 10 g/dl, 12 g/dl and gender-specific (male 13 g/dl, female 12 g/dl) subgroups with *I*^*2*^ of 35.5, 55.3 and 4.2%, respectively (Fig. 3).

**Fig. 4:**
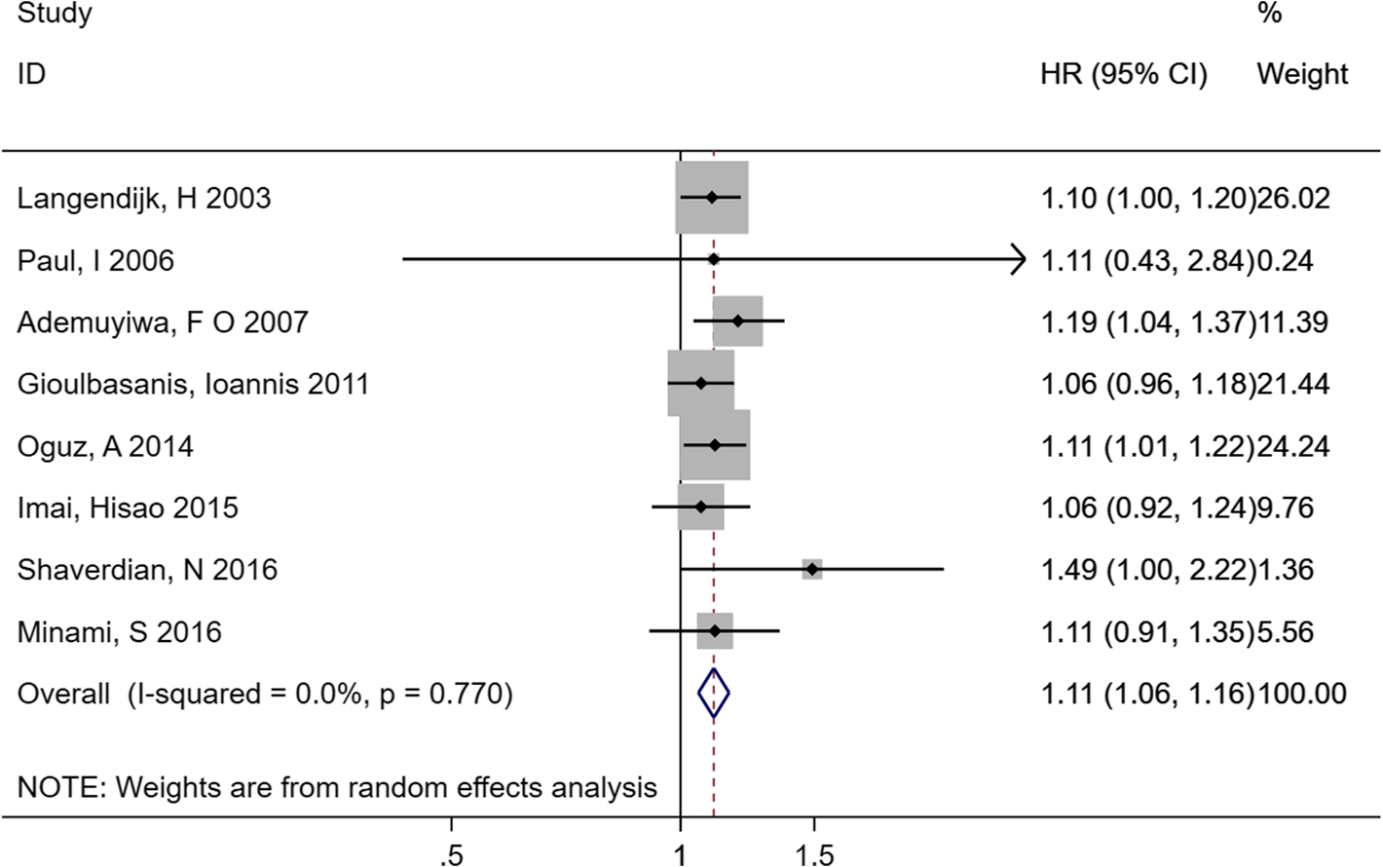
Forest plot and pooled HR and 95% CI for the association between pretreatment Hb level (continuous variable) and OS in patients with lung cancer. The pooled HR indicated that decreased Hb was related to the poor OS. *HR* hazard ratios, *CI* confidence interval, *Hb* hemoglobin

Eight cohorts analysed the Hb level data as a continuous variable and evaluated the correlation between pretreatment Hb level and OS. We found that a decreased Hb level was significantly related to OS (HR 1.11, 95% CI 1.06–1.16) with no significant heterogeneity (*I*^*2*^ = 0.0%, *p* = 0.770) (Fig. 4).

### Prognostic impact of decreased Hb on patients with NSCLC

**Fig. 5:**
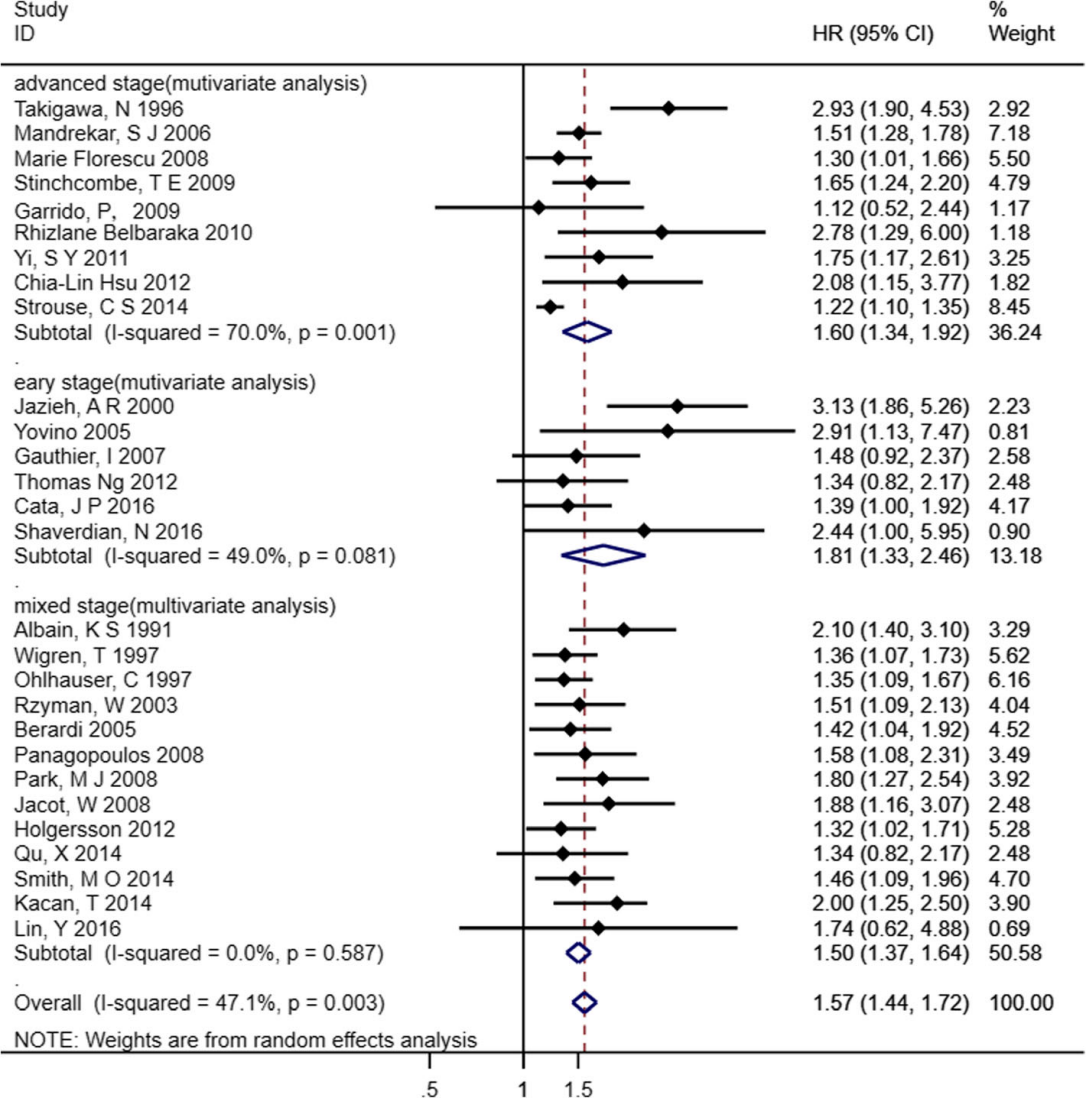
Forest plot and pooled HR and 95% CI for OS in patients with NSCLC: pretreatment decreased Hb vs. normal Hb. The pooled HR for OS indicated that pretreatment decreased Hb level had a negative impact on survival of patients with NSCLC both in early stage and advanced stage. *NSCLC* non-small cell lung cancer, *HR* hazard ratios, *OS* overall survival, *CI* confidence interval, *Hb* hemoglobin

Twenty-eight studies evaluated the prognostic impact of decreased Hb (categorical variable: decreased Hb vs. normal Hb) on NSCLC in multivariate analyses. We found that decreased Hb was a poor prognostic marker for OS (HR 1.57, 95% CI 1.44–1.72) with moderate heterogeneity (*I*^2^ = 47.1%, *p* = 0.003). Subgroup analyses were conducted according to tumour stage. The result indicated that decreased Hb had a prognostic impact on OS for patients in early stage (HR 1.81, 95% CI 1.33–2.46), advanced stage (HR 1.60, 95% CI 1.34–1.92) and both (HR 1.50, 95% CI 1.37–1.64), although the heterogeneity was significant in the advanced stage subgroup (*I*^*2*^ = 70%, *p* = 0.001) (Fig. 5).

### Prognostic impact of decreased Hb on patients with SCLC

**Fig. 6:**
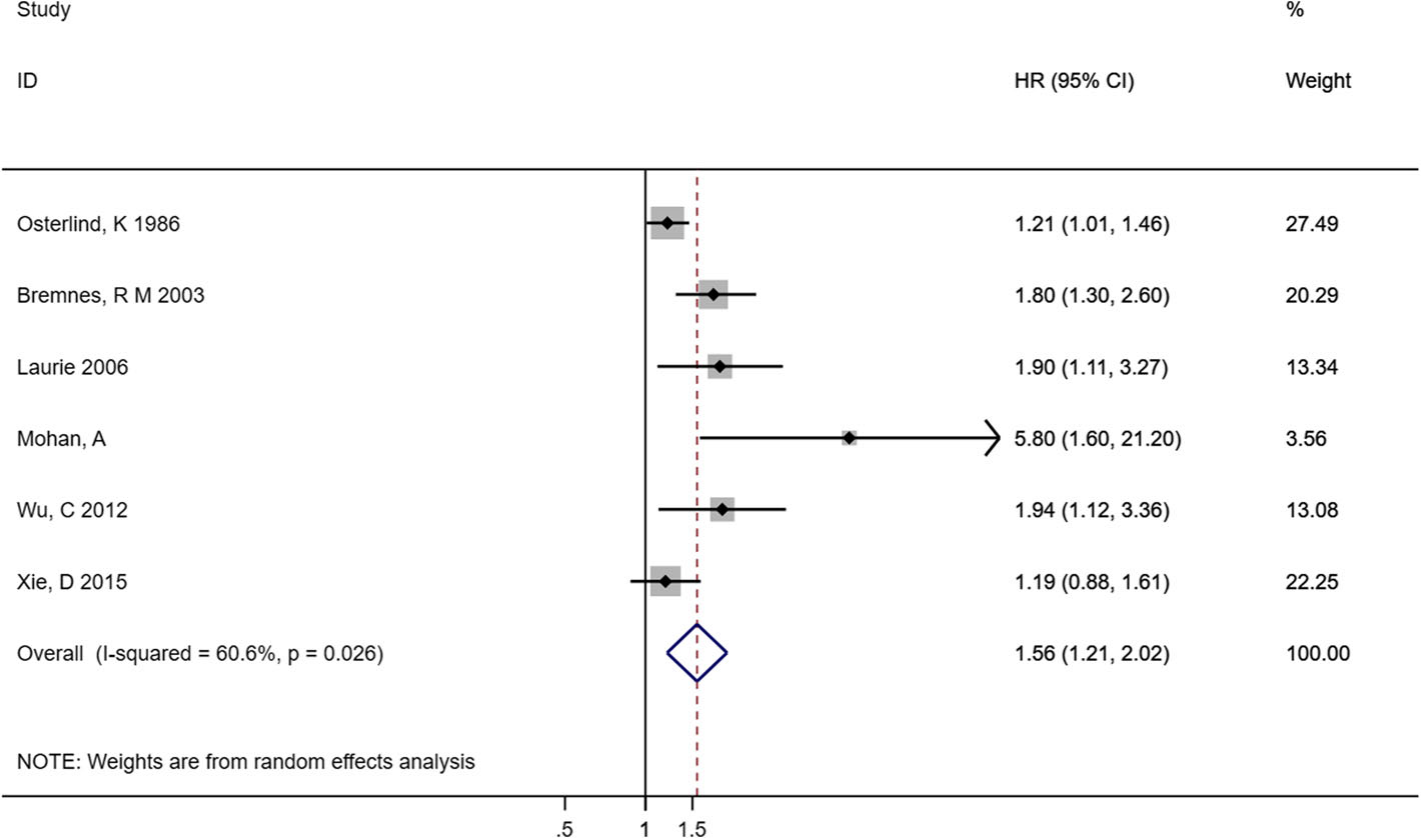
Forest plot and pooled HR and 95% CI for OS in patients with SCLC: pretreatment decreased Hb vs. normal Hb. The pooled HR for OS showed decreased Hb level was associated with shorter OS. *SCLC* small cell lung cancer, *HR* hazard ratios, *OS* overall survival, *CI* confidence interval, *Hb* hemoglobin

Six cohorts with 3203 cases reported the data of pretreatment Hb (categorical variable: decreased Hb vs. normal Hb) and OS in patients with SCLC. The pooled HR from the 6 cohorts showed that patients with decreased Hb were associated with shorter OS (HR 1.56, 95% CI 1.21–2.02), although there was significant heterogeneity among the studies (*I*^*2*^ = 60.6%, *p* = 0.026) (Fig. 6).

### DFS and decreased Hb

**Fig. 7:**
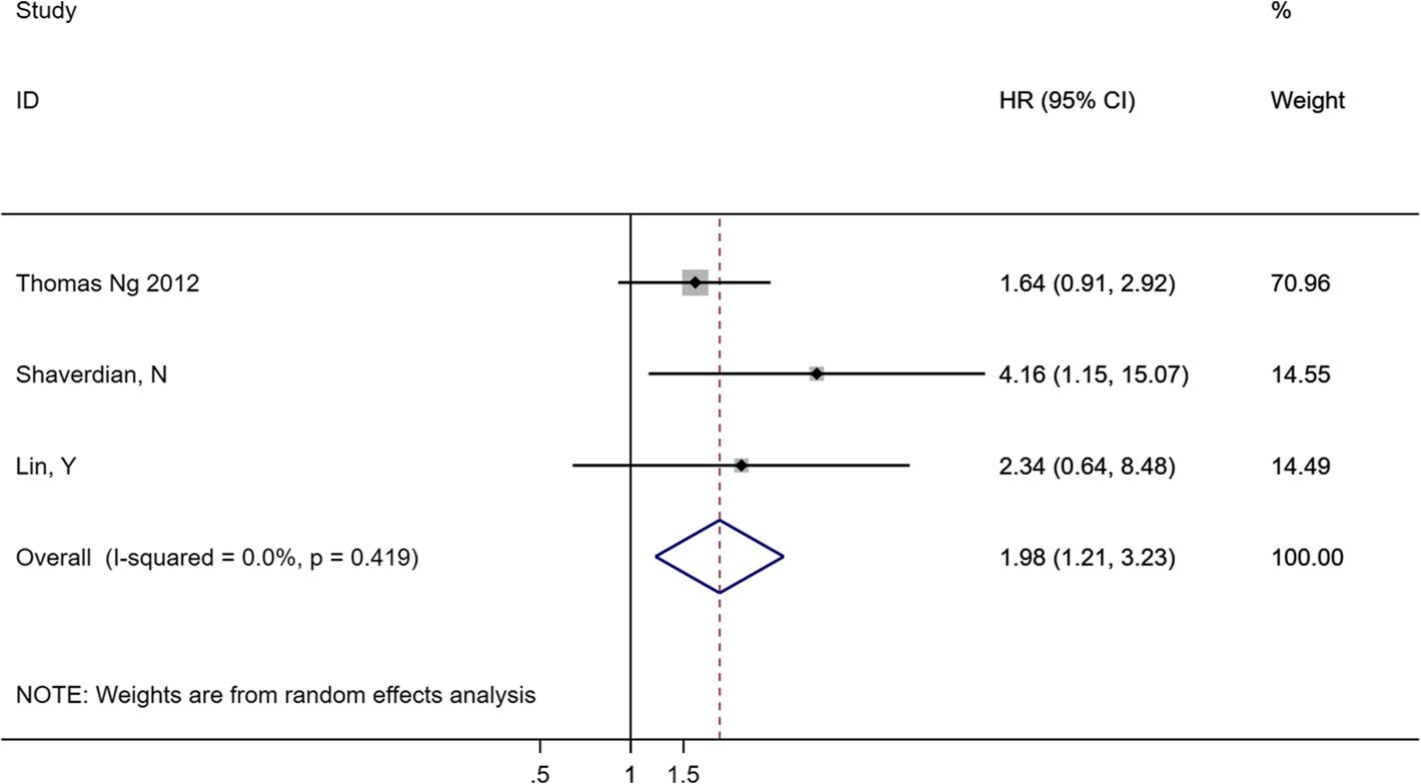
Forest plot and pooled HR and 95% CI for DFS in patients with lung cancer: pretreatment decreased Hb vs. normal Hb. The pooled HR for DFS showed pretreatment decreased Hb level was associated with shorter DFS. *HR* hazard ratios, *DFS* disease-free survival, *CI* confidence interval, *Hb* hemoglobin

Three studies presented the data from their investigation of pretreatment Hb (categorical variable: decreased Hb vs. normal Hb) and DFS. The combined data suggested that decreased pretreatment Hb was significantly correlated with DFS, with a pooled HR estimate of 1.98 (95% CI 1.21–3.23) and no heterogeneity (*I*^*2*^ = 0.0%, *P* = 0.419) (Fig. 7).

### RFS and decreased Hb

**Fig. 8:**
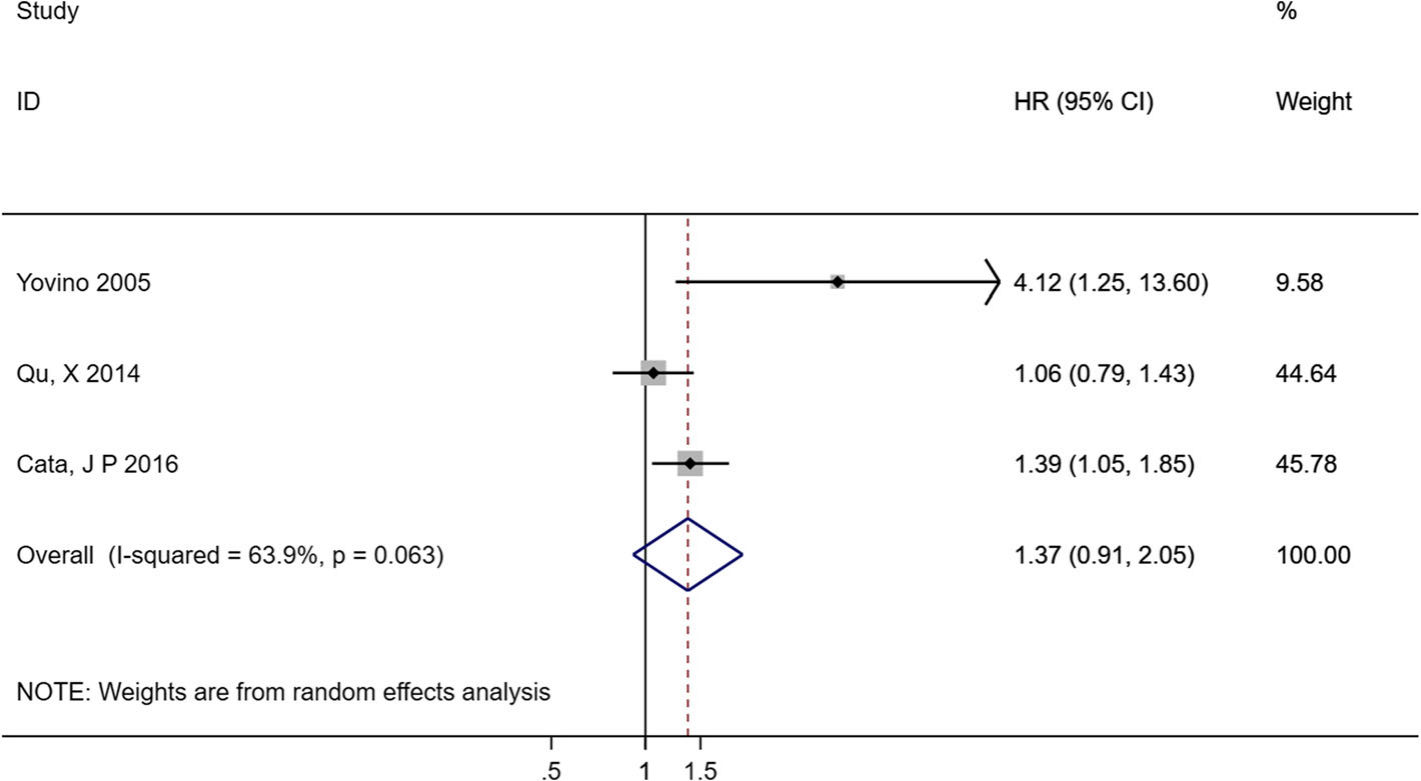
Forest plot and pooled HR and 95% CI for RFS in patients with lung cancer: pretreatment decreased Hb vs. normal Hb. The pooled HR for RFS showed pretreatment decreased Hb level was not significantly associated with shorter RFS. *HR* hazard ratios, *RFS* relapse-free survival, *CI* confidence interval, *Hb* hemoglobin

Three studies reported the correlation between RFS and decreased Hb (categorical variable: decreased Hb vs. normal Hb). Interestingly, the pooled HR indicated that decreased pretreatment Hb was not significantly associated with shorter RFS (HR 1.37, 95% CI 0.91–2.05), and the heterogeneity was not significant (*I*^*2*^ = 63.9%, *p* = 0.063) (Fig. 8).

### Meta-regression analyses

To further explore the potential causes of the heterogeneity, treatment method and sample size were used to conduct meta regression after the subgroup analysis. The results showed that these two factors were not the source of heterogeneity.

### Sensitivity analysis and publication bias

In our meta-analysis, the Begg’s funnel plot and Egger’s indicator test were used to evaluate potential publication bias for OS. As our results show in Additional file 1**:** Figure S1 and Additional file 2**:** Figure S2, both the Begg’s funnel plot and Egger’s publication bias plot indicate the existence of publication bias among the included studies (*p* < 0.001). Interestingly, sensitivity analysis revealed that none of the HR point estimates lay outside the 95% CI of the pooled analysis, which confirmed that our results were stable and reliable.

## Discussion

Lung cancer is a leading cause of cancer death worldwide with about 15% of 5-year survival rate [1]. It is well known that the TNM system has played an important role in the evaluation of clinical outcome and the decision-making process of selecting effective therapies. However, the complexity of its pathogenic mechanism means that the progression and prognosis of cancer can be caused by many factors. Patients with the same pathological stage often present with different outcomes, which suggests that the TNM system alone cannot precisely predict the survival of patients with lung cancer. Moreover, the TNM stage should be confirmed by biopsy; therefore, it is difficult to track stage changes in the process of cancer progression. Peripheral blood samples are easily obtained by nurses with less clinical practice cost. The current viewpoint considers that some haematological biomarkers are related to the prognosis of cancers, including the neutrophil to lymphocyte ratio [73], leucocyte [74], platelet [75], white blood cell [54] and Hb levels [76] before treatment. However, the prognostic value of the Hb level in patients with lung cancer remains controversial.

Many researchers aimed to develop a new evaluation or model to predict the expected lifetime of patients with lung cancer [66, 77]. The creation of such instruments requires to identify the survival prediction value of pretreatment peripheral blood markers and other clinicopathological factors. Hb is an important hematological marker to predict the survival in patient with cancer. However, the prognostic value of decreased pretreatment Hb level on survival remains controversial. This systematic review and meta-analysis are the first evidence-based research to determine the prognostic impact of decreased pretreatment Hb on the OS, DFS and RFS of patients with lung cancer, which can make contributions to the personalized treatment programs.

In this systematic review with meta-analyses of 55 eligible studies, we first evaluated the relationship between decreased Hb and OS in patients with lung cancer. The results showed that patients with a Hb reduction at the time of diagnosis or before treatment were significantly associated with poor OS in both univariate and multivariate analysis. A significant heterogeneity was observed, but the pooled HRs were stable when deleting each study one by one. Thus, a random effect model was selected to analyse the pooled HR, and subgroup analyses and meta-regression were conducted. We also found that there were more studies of the prognostic value of decreased Hb in patients with NSCLC than in patients with SCLC. However, similar results confirmed that a decreased Hb level was a negative prognostic factor for OS in both patients with NSCLC and SCLC. Other survival indicators were also applied to this meta-analysis. Interestingly, different results were found for the prognostic value of preoperative Hb on DFS and RFS. As shown in Fig. 7 and Fig. 8, a decreased pretreatment Hb level was significantly associated with poor DFS, while in three studies addressing RFS, the pooled HR indicated that the prognostic value of Hb was not significant. In the pooled analysis of the continuous variable Hb level and OS, it can be postulated that, even if the Hb level was in the normal range, a lower Hb level was significantly associated with worse survival in patients with lung cancer.

The cause of Hb degradation is multifactorial and often relates to other comorbidities. It is reported that the systemic inflammatory responses from tumour cells strongly correlate with cancer progression and malignant transformation [78]. Specifically, interleukin-6 (IL-6) is an important inducer of the production of hepcidin, which is involved in iron metabolism. Elevated hepcidin levels lead to reductions in serum iron levels and result in decreased Hb [79]. It should be noted that higher hepcidin levels have been detected in patients with more aggressive diseases [79]. The mechanism underlying the prognostic value of decreased Hb in patients with lung cancer can be explained from several perspectives. Hb reduction contributes to hypoxia of tumour cells, which then stimulates tumour growth and increases the resistance of tumour cells to radiotherapy and chemotherapy by regulating the gene expression and cell-cycle position, subsequently causing progression of cancer and shorter survival [80].

Two principal options for the management of decreased Hb have been proposed by previous studies, including the use of erythropoiesis-stimulating agents (ESAs) and blood transfusion [81]. ESAs could increase Hb levels and reduce transfusion requirements [82]. However, a meta-analysis of randomized controlled trials showed that the use of ESAs was associated with an increased risk of developing venous thromboembolism in cancer patients [83]. Therefore, the safety of treatment with ESAs in cancer patients still needs to be considered. Blood transfusion is effective for correcting Hb decline and improving symptoms or signs induced by decreased Hb in patients with cancer. However, it has been reported that perioperative blood transfusion was associated with an increased recurrence of lung cancer due to transfusion-related immunomodulation [84]. Overall, further studies are needed to investigate how to effectively manage decreased Hb in patients with lung cancer.

There are several limitations presented in this meta-analysis. First, the recruited data were extracted from observational studies, most of which were retrospective cohort studies; only two studies were based on prospective cohorts. Additionally, the cut-off values defining decreased Hb in our meta-analysis were not consistent, 10 g/dl, 11 g/dl, 11.5 g/dl, 11.6 g/dl, 12 g/dl, 12.5 g/dl, 12.7 g/dl, 12.8 g/dl, 13 g/dl, 13.1 g/dl, 13.2 g/dl, 13.6 g/dl, 14 g/dl and 14.6 g/dl. This confounder may influence the outcomes. To strengthen the power of our results, studies with 10 g/dl, 11 g/dl, 12 g/dl and gender-specific (male, 13 g/dl; female, 12 g/dl) cut-off values were analysed in the meta-analysis and similar results were obtained, specifically that decreased Hb was significantly associated with poor OS in patients with lung cancer. In fact, pooled results of the analysis of the continuous variable Hb and OS suggested that, even when the Hb level was within the normal range, lower Hb levels may predict the poor outcomes of survival and still need attention. Third, mild to moderate potential heterogeneity may exist between the included studies. We evaluated the prognostic value of Hb in NSCLC and SCLC separately. Subgroup analyses and meta-regression were conducted to detect the source of heterogeneity. Although the results suggested that region, subtype of lung cancer, treatment method and cut-off value were not the source of heterogeneity, there were still different features between the trials, and these features may be highly correlated and were not easily detected. Fourth, previous systematic review and meta-analysis showed that blood transfusions adversely affected cancer survival [85]. It was reported that the significant correlation between low Hb level and poor OS may be due to erythropoietin treatment or blood transfusion before surgery [86]. In our meta-analysis, since the data on how many patients received a blood transfusion during their survival time were not available, we cannot determine whether decreased pretreatment Hb or blood transfusion was the major factor of survival. However, this meta-analysis still explained the negative impact of decreased Hb on survival in patients with lung cancer to some extent. Further research on whether the decreased Hb levels before treatment directly affect the survival of patients with lung cancer, rather than blood transfusions, remains to be conducted. Fifth, there was significant publication bias for the correlation between decreased pretreatment Hb and OS in patients with lung cancer given the results of Begg’s funnel plot and the Egger’s test. The number of included articles was sufficient, but some of the baseline characteristics of the recruited studies differed in some confounders (gender, sample size, treatment, period of follow-up, etc.), which may contribute to the bias. We improved the stability of our estimation of the impact of decreased Hb on the prognosis of lung cancer by using sensitivity analysis. However, a publication bias still existed for the estimated pooled HR on OS. Finally, it was reported that not only did a lower Hb level lead to poor prognosis but abnormally elevated Hb did as well [87]. In this meta-analysis, we only focused on the impact of decreased Hb on survival, and further investigation and trials about the prognostic effects of abnormally elevated Hb on the survival of patients with lung cancer are needed.

## Conclusion

In conclusion, our findings suggested that a decreased Hb level before treatment was a prognostic indicator of shorter OS and DFS both in patients with NSCLC and SCLC. The Hb level, an economical and readily available marker, might serve as an indicator for survival prediction, risk stratification and treatment selection. However, because of the limitation of our current study, additional large prospective cohorts and experimental trials are needed to confirm Hb level as an independent predictor of prognosis in patients with lung cancer. Additionally, targeting the correction of pretreatment Hb degradation may be an effective strategy to increase the survival rate of patients with lung cancer.

## Additional files

Additional file 1: **Figure S1.** Begg’s funnel plot for included studies. (JPG 75 kb)

Additional file 2: **Figure S2.** Egger’s indicator test for included studies (JPG 69 kb)

## Abbreviations

CI: Confidence interval
DFS: Disease-free survival
EFS: Event-free survival
Hb: Hemoglobin
HR: Hazard ratio
MV: Multivariate
NCCN: National Comprehensive Cancer Network
NOS: Newcastle-Ottawa scale
NR: Not reported
NSCLC: Non-small cell lung cancer
OS: Overall survival
PFS: Progress-free survival
RFS: Relapse-free survival
SCLC: Small cell lung cancer
TNM: Tumor-node metastasis
TTP: Time to progression
UV: Univariate
