# Supplementary figures and images for: The prognostic impact of decreased pretreatment haemoglobin level on the survival of patients with lung cancer: a systematic review and meta-analysis
Source: BMC Cancer. 2018 Dec 10;18:1235. doi: 10.1186/s12885-018-5136-5 (PMC6288911; doi:10.1186/s12885-018-5136-5)

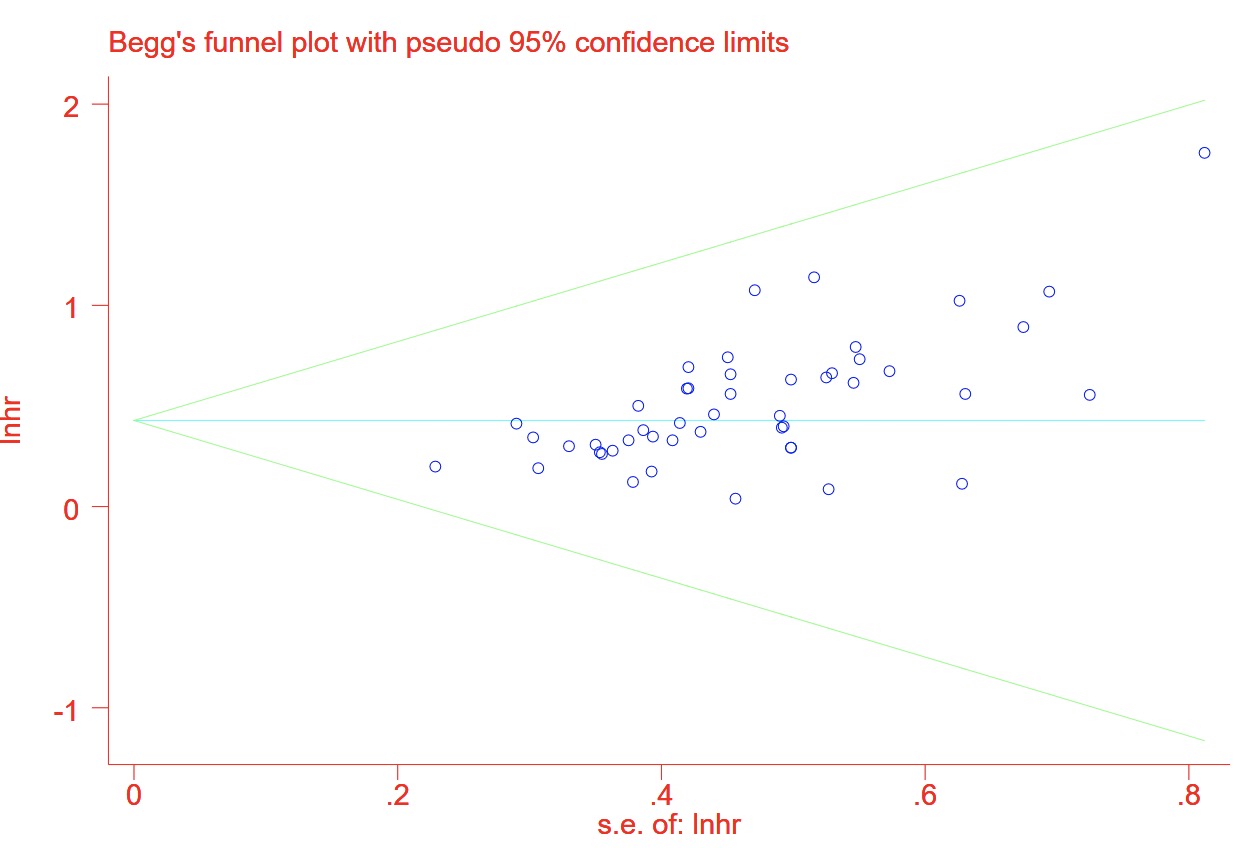

Supplement: Supplementary file 1 — Figure S1. Begg’s funnel plot for included studies. (JPG 75 kb) [file 12885_2018_5136_MOESM1_ESM.jpg]

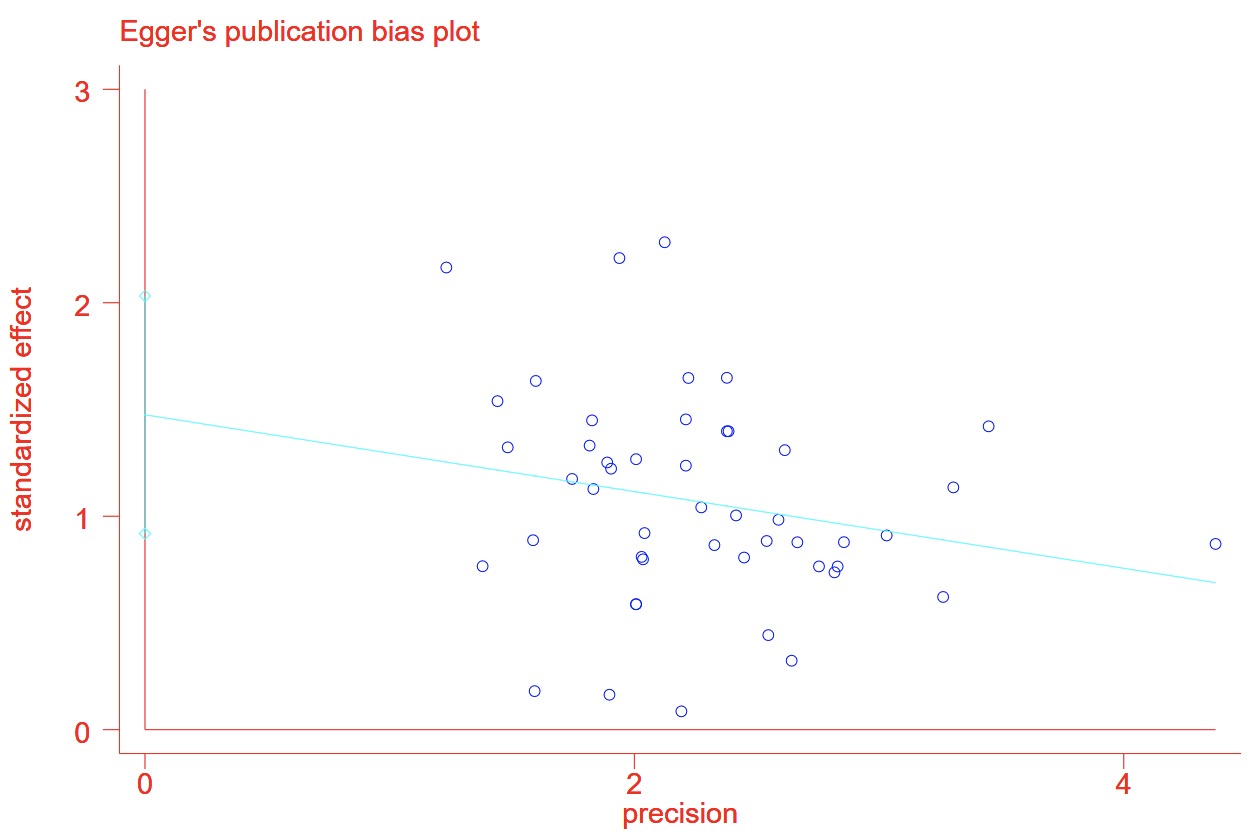

Supplement: Supplementary file 2 — Figure S2. Egger’s indicator test for included studies (JPG 69 kb) [file 12885_2018_5136_MOESM2_ESM.jpg]
